# N-Acetylcysteine Treats Spinal Cord Injury by Inhibiting Astrocyte Proliferation

**DOI:** 10.1155/2024/6624283

**Published:** 2024-05-29

**Authors:** Dong Zhang, Chaoxi Qin, Fei Meng, Xiaopeng Han, Xing Guo

**Affiliations:** ^1^Changzhi Medical College, No. 161, Jiefang East Street, Changzhi 046000, China; ^2^Department of Orthopedics, Jincheng General Hospital, China Kangping Street, Beishidian Town, Jincheng 048006, China

## Abstract

Astrocyte proliferation commonly occurs after spinal cord injury (SCI). N-Acetylcysteine (NAC) has a regulatory effect on many diseases. In this study, we investigated the effect and underlying mechanism of NAC on astrocytes in SCI. We isolated rat primary astrocytes and stimulated with lipopolysaccharide to induce cell proliferation and degeneration. A rat model of SCI was also established, and the Basso–Beattie–Bresnahan score was determined. The localization of glial fibrillary acidic protein in the cells and tissues was determined using TUNEL staining and immunofluorescence, while that of connexin 43 was assessed via immunofluorescence. Pathological changes associated with SCI were detected using hematoxylin and eosin staining, and inflammatory factors were detected using enzyme-linked immunosorbent assay. Additionally, JAK/STAT expression was evaluated using western blotting and quantitative reverse transcription polymerase chain reaction. NAC downregulated the glial fibrillary acidic protein abundance and connexin 43 in reactive astrocytes and SCI rat models while inhibiting the abundance of secreted proteins DSPG, HSPG, KSPG, tenascin C, vimentin, CSPG, ephrin-B2, and nestin. NAC also regulated the JAK/STAT signaling pathway by downregulating the expression of JAK2, STAT5, STAT3, STAT1, PIM1, NFATc1, COL1, COL3, TGF-*β*, SMAD1, CTGF, CyCD1, and CDK4, thus alleviating SCI. Finally, NAC exhibited durable effects, with no SCI recurrence within 60 days. Therefore, NAC relieves SCI by inhibiting the proliferation of reactive astrocytes and suppressing the expression of secretory and JAK/STAT pathway proteins.

## 1. Introduction

Spinal cord injury (SCI) can be classified as traumatic or nontraumatic. Traumatic SCI is defined as trauma caused by an external physical impact; degeneration is a complication of SCI that can lead to limb dysfunction [1]. In contrast, nontraumatic SCI occurs in acute or chronic diseases and results in serious injury [2]. Most SCIs damage systems that regulate urinary and bowel control, respiration, heart rate, and blood pressure. Methylprednisolone sodium succinate is approved by the Food and Drug Administration for SCI treatment. The administration of this drug 8 hr after SCI attenuates secondary injury. However, as the immune system of patients with SCI is weakened, further suppressing the immune response can increase the incidence of infections and other diseases [3].The internal pressure of the spinal cord parenchyma increases following SCI, resulting in ischemia and hypoxia and ultimately accelerating the cascade reaction. Hence, early surgical decompression is associated with the recovery of neurological function in patients with SCI [4]. Indeed, surgical decompression can reduce the incidence of secondary injuries; however, the safety and effectiveness of decompression surgery and the timing of surgery often vary [5].

Astrocytes are the most abundant “glial” cells in the central nervous system [6]. Following SCI, astrocytes surrounding the injury become altered via proliferative hypertrophy and protrusion extension. Destruction of the spinal cord produces cell fragments associated with pattern recognition receptors on inflammatory cells, activation of which induces chemokine and cytokine release by astrocytes [7]. In this way, astrocytes can aid in tissue repair, while scar-shaped astrocytes ultimately form a glial scar, which prevents axonal regeneration [8]. Functionally, astrocyte scars protect nearby surviving neural tissue and reduce the amplification of destructive inflammatory factors [9]. More specifically, the neuroinflammatory response in SCI induces a series of cellular and molecular events, including astrocyte activation and infiltration of peripheral blood macrophages [10]. Overactivated reactive astrocytes can transform into scar-forming astrocytes; the formation of a glial scar impedes SCI recovery [11]. These findings have prompted more extensive research on the role of astrocytes in SCI [11, 12, 13]. However, the role of astrocytes in SCI still needs to be further explored.

The JAK/STAT signaling pathway is regulated by various cytokines and plays a dual role in cell proliferation and cell death. This pathway serves as an activator of various cytokines, leading to cell growth and death [14]. Moreover, the JAK/STAT pathway is one of the most important mechanisms underlying glial cell fate determination and is impacted by astrocyte hyperactivation [15]. Furthermore, this pathway exerts neuroprotective effects on astrocytes [16]. According to Song et al. [17], mullein isoflavones attenuate oxidative damage in spinal cord astrocytes by mediating the GP130/JAK/STAT pathway. Meanwhile, Lee et al. [15] reported that astrocyte-derived ciliary neurotrophic factor protects retinal ganglion cells through the JAK/STAT pathway.

N-Acetylcysteine (NAC) is a sulfur-containing N-acetyl derivative synthesized from the endogenous amino acid L-cysteine [18] that scavenges reactive oxygen species. NAC exerts inhibitory protective effects in various diseases, including Parkinson's [19], Alzheimer's disease [20], liver disease [21], coronavirus disease [22], and severe acute respiratory syndrome coronavirus-2 [23]. Accordingly, NAC has been approved by the US Food and Drug Administration as a safe and well-tolerated drug for treating several diseases. NAC also protects against traumatic SCI [24]; NAC combination therapy elicits a synergistic neuroprotective effect [25, 26]. Additionally, NAC lessens SCI by regulating inflammation and apoptosis after initial decompression surgery in rats [27]. However, the regulatory mechanism of NAC on astrocytes in SCI has not been systematically investigated.

We hypothesize that NAC can repair SCI in rats and inhibit astrocyte proliferation after SCI. To test this hypothesis, we examine the role and effect of NAC on astrocytes *in vivo* and *in vitro* to provide a new therapeutic approach for SCI.

## 2. Materials and Methods

### 2.1. Separation of Primary Astrocytes

Neonatal rats, purchased from the Hubei Animal Research Center (Hubei, China), were anesthetized via intraperitoneal injection of 1% pentobarbital sodium (100 mg/kg). The brain tissue of the neonatal rats was removed under sterile conditions, and the cerebral cortex was isolated. Cerebral cortex tissues were collected for transmission electron microscopy (TEM) analysis. Dulbecco's modified Eagle's medium (HyClone, Logan, UT, USA) was added to the remaining cerebral cortex tissues. The tissues were cut into 1 mm^3^ pieces, placed in media comprising 0.25% trypsin and 0.2% collagenase (Bioswamp, Wuhan, China), and digested at 37°C for 30 min; subsequently, DMEM with 10% fetal bovine serum (FBS; Gibco, Grand Island, NY, USA) was added to stop the digestion [28]. Undigested tissue was removed using 200 mesh sieves and centrifuged at 888 × *g* for 10 min; this step was repeated thrice. Primary astrocytes were obtained by removing fibroblasts using differentiation and planning methods. Primary astrocytes were cultured in DMEM with 10% FBS in a 5% CO_2_ atmosphere at 37°C. All animal experiments were conducted in accordance with the relevant provisions of the Ethical Certificate of Experimental Animals of the Animal Administration Commission of Hubei Province (license number for laboratory animals: SYXK (E) 2018-0104; Certificate No.: 11401500039868.

### 2.2. *In Vivo* Model Construction and Identification

Fifty Sprague–Dawley rats were purchased from the Hubei Animal Research Center; 40 rats were anesthetized by injecting 1% sodium pentobarbital (30–40 mg/kg) intraperitoneally [29]. A posterior median longitudinal incision, approximately 2.5 cm in length, was centered on the T13 spinous process; the T13 vertebral plate was occluded during the procedure using a handheld X-ray machine to confirm the correct position. A 5/0 silk thread was reversed, and the nose end of a small circular needle was passed through the potential gap between the anterior edge of the dural sac and the posterior wall of the vertebral body. Finally, the thread was cut to remove the needle. One strand of the thread was used as the measuring thread, and the other served as the loop tie thread. Additionally, one end of the loop tie thread was left long and dry to facilitate marking. The circumference of the dural sac was measured under a microscope (10x magnification) using a measuring wire loop. The loop was measured as tightly as possible without preventing the filling of the blood vessels on the dorsal side of the spinal dural sac. The dural sac was removed after its circumference (C1) was measured. The length of the dural sac was measured using a waterproof digital vernier caliper (accurate to 0.01 mm), and the cross-sectional compression of the dural sac to 70% of the original cross-sectional area was obtained algebraically (C2 = C1*⁣*^*∗*^). The circumference (C2) was obtained through algebraic calculation (C2 = C1*⁣*^*∗*^). After precisely setting the C2 length with the digital vernier caliper, the length was marked on the percussion line with a black oil-based marker. Subsequently, the paravertebral muscles and fascia were continuously sutured after ligation; the skin was disinfected, and the incision was sutured. We used the Basso–Beattie–Bresnahan score (BBB) [30] to assess the locomotor ability of rats.

The rats were randomly divided into five groups (*n* = 10/group): control group (rat laminectomy and personalized measurement of dural sac circumference), De-8h group (rats with simple decompression 8 hr after spinal cord cerclage), De-48h group (simple decompression 48 hr after rat spinal cord cerclage), De-8h+NAC group (decompression 8 hr after rat spinal cord cerclage, 50 mg/kg NAC intraperitoneal injection [31] every other injection once daily for 2 weeks), and De-48h+NAC group (decompression of rats 48 hr after spinal cord cerclage, 50 mg/kg NAC intraperitoneal injection every other day for 2 weeks). Five rats were sacrificed on postoperative days 3 and 60. Spinal cord tissues from some rats were collected for paraffin sectioning and stored in 4% paraformaldehyde; those from the remaining rats were cryopreserved.

### 2.3. Immunofluorescence

Cells and spinal cord tissues were fixed in 4% paraformaldehyde (Sinopharm, Shanghai, China) and permeabilized with 0.5% Triton X-100 (Bioswamp) at 25°C for 20 min. The cells were blocked with 5% bovine albumin at 37°C for 1 hr. Subsequently, primary antibodies against glial fibrillary acidic protein (GFAP) (1 : 100, MAB50947, Bioswamp) and connexin 43 (CX43) (1 : 100, PAB30808, Bioswamp) were added and incubated in wet boxes overnight at 4°C. Alexa Fluor 594-conjugated goat antimouse IgG (1 : 200, Bioswamp) and Alexa Fluor 488-conjugated goat antimouse IgG (1 : 200, SAB51370, Bioswamp) secondary antibodies were added and incubated for 1 hr at 37°C. Next, an antifluorescence quenching blocking solution containing 4,6-diamidino-2-phenylindole (Bioswamp) was added dropwise. Images were captured using a Nikon C2 laser confocal microscope (Tokyo, Japan) and analyzed using a Nikon Elements Advanced Research image analyzer.

### 2.4. TEM

Cerebral cortex tissues were fixed with 2.5% glutaraldehyde for 30 min, washed thrice with 0.1 mol/L phosphate-buffered saline (PBS) for 10 min each, transferred and fixed in 1% osmic acid for 1 hr, and washed thrice with 0.1 mol/L PBS for 10 min each. The cells were sequentially transferred into 50%, 70%, 90%, and 100% ethanol for 5 min each, immersed in acetone-to-epoxy resin (1 : 1) solution, and embedded for 6 hr. The tissues were sliced into 60 nm sections, stained with uranyl acetate in the dark for 20 min, washed with double-distilled water, transferred to lead citrate for 15 min in the dark, and washed with double-distilled water. The sections were observed using TEM (H-7700, Hitachi, Tokyo, Japan). All reagents for TEM were purchased from Beijing Zhongjing Technology Co., Ltd. (Beijing, China).

### 2.5. *In Vitro* Model Construction and Identification

Primary astrocytes were divided into five groups. The normal control (NC) cells were treated with LPS-free medium; the lipopolysaccharide (LPS) cells were treated with LPS-containing (1 *μ*g/mL) [32] medium) and incubated for 12–24 hr after dosing; the WP1066 cells were treated with LPS (1 *μ*g/mL), incubated for 12–24 hr before changing the solution, washed twice with PBS, and treated with pre-1 *μ*M [33] JAK inhibitor; cells in the NAC1, NAC2, NAC3, and NAC4 groups were treated with LPS (1 *μ*g/mL), incubated for 12–24 hr before changing the media, washed twice with PBS, and treated with different concentrations of NAC (0.5, 1, 2.5, or 5 mM, respectively) for 24 hr. TEM revealed the occurrence of astrocyte hyperplasia. GFAP + GFAP-CX43 was detected using immunofluorescence, and astrocyte proliferation was assessed via TEM.

### 2.6. Enzyme-Linked Immunosorbent Assay (ELISA)

The levels of interleukin (IL)-1*α* (IL-1*α*, RA20610), tumor necrosis factor (TNF-*α*, RA20035), dermatan sulfate proteoglycan (DSPG, RA21202), heparan sulfate proteoglycan (HSPG, RA21423), keratan sulfate proteoglycan (KSPG, RA21203), tenascin C (RA21421), vimentin (RA21032), chondroitin sulfate proteoglycan (CSPG, RA21201), ephrin-B2 (RA21422), and nestin (RA21304) were detected in serum and spinal cord tissue using ELISA kits (Bioswamp). Assays were performed according to the manufacturer's protocol, and the optical density was measured at 45 nm. In brief, RIPA buffer (Bioswamp) was added to the samples, homogenized, and centrifuged to collect the supernatant. Next, 100 *μ*L of standard was added to 100 *μ*L of standard diluent, mixed, and repeated six times. We then added the sample and the corresponding antibody to a blank well of the ELISA plate, mixed the solution, added horseradish peroxidase-conjugate reagent, and incubated the sample at 37°C for 30 min. The liquid on the plate was discarded. The plate was dried using rotation, after which the washing solution was added, and the plate was allowed to stand for 30 s; these steps were repeated five times. Chromogenic agent was added, and the plate was protected from light and incubated at 37°C for 10 min. Termination solution was added, and the optical density was measured at a wavelength of 450 nm using a microplate reader (Mettler Toledo, Columbus, OH, USA). All reagents were provided in the ELISA kits.

### 2.7. Western Blotting

RIPA buffer (Bioswamp) was added to the spinal cord tissues and homogenized to isolate the proteins. The protein concentration was detected using a bicinchoninic acid assay (BCA) kit (Bioswamp). Samples were loaded at a volume of 20 *μ*g per well, and the electrophoresis voltage was controlled at 80 V for 40 min and then adjusted to 120 V for 50 min. Proteins were transferred onto polyvinylidene fluoride membranes (Millipore, Billerica, MA, USA) via wet transfer at 90 V for 50 min. The membranes were blocked with 5% skim milk powder at 25°C for 2 hr and incubated with the following primary antibodies individually at 4°C overnight: Pim-1 proto-oncogene (PIM1, 1 : 500, ab54503, Abcam, Cambridge, UK), nuclear factor of activated T cells 1 (NFATc1, 1 : 1,000, PAB32461, Bioswamp), collagen I (COL1, 1 : 1,000, PAB330493, Bioswamp), collagen III (COL3, 1 : 1,000, ab23445, Abcam), transforming growth factor-*Β* (TGF-*β*, 1 : 2,000, ab170874, Abcam), Smad family member 1 (SMAD1, 1 : 5,000, ab108965, Abcam), cellular communication network factor 2 (CTGF, 1 : 1,000, ab6992, Abcam), cyclin D1 (CYCD1, 1 : 250, ab224044, Abcam), cyclin-dependent kinase 4 (CDK4, 1 : 5,000, ab108357, Abcam), Janus kinase 2 (JAK2, 1 : 5,000, ab108596, Abcam), p-JAK2 (1 : 5,000, ab32101, Abcam), signal transducer and activator of transcription 5 (STAT5, 1 : 1,000, ab16276, Abcam), p-STAT5 (1 : 1,000, ab32364, Abcam), p-STAT3 (1 : 1,000, ab76315, Abcam), STAT3 (1 : 5,000, ab119352, Abcam), p-STAT1 (1 : 1,000, ab30645, Abcam), STAT1 (1 : 1,000, ab92506, Abcam), and glyceraldehyde-3-phosphate dehydrogenase (GAPDH, 1 : 10,000, PAB160009, Bioswamp). The membrane was washed thrice with 0.05% Tween-20 in PBS (Solarbio, Beijing, China) for 5 min each. The secondary antibody (1 : 20,000) was incubated for 1 hr at 25°C after washing the membrane thrice. After 1 hr, the membrane was removed, and 0.05% Tween-20 in PBS was added to wash the membrane thrice for 5 min each. The membrane was removed, and the enhanced chemiluminescence solution was added and placed in a fully automated chemiluminescence instrument (TIAN NENG, China). The grayscale values of the relevant bands were read using Tanon GIS software (Shanghai, China).

### 2.8. Quantitative Real-Time PCR

Trizol (1 mL; Ambion, Austin, TX, USA) was added to the cells and spinal cord tissue samples, homogenized for 20 s, and immediately placed on ice. This sample was centrifuged at 12,000 × *g* for 10 min; the supernatant was removed, mixed with 200 *μ*L of chloroform, and allowed to stand for 2 min. The supernatant was centrifuged at 12,000 × *g* for 10 min, mixed with isopropyl alcohol, allowed to stand for 15 min at 4°C, and centrifuged at 12,000 × *g* for 15 min before being discarded. Subsequently, 1 mL of 75% ethanol was added and centrifuged at 7,500 × *g* at 4°C for 5 min; 1 mL of 75% ethanol was added, and the sample was centrifuged at 7,500 × *g* at 4°C for 5 min and then dried at room temperature for 10 min. DNase/RNase-free water (40 *μ*L; Solarbio) was added to dissolve the precipitate. cDNA was reverse-transcribed using a kit reaction system (TAKARA, Shiga, Japan), and PCR amplification was performed using cDNA as the template as follows: 95°C for 3 min, 95°C for 5 s, 56°C for 10 s, and 72°C for 25 s, 39 cycles, followed by 65°C for 5 s and 95°C for 50 s. The results were analyzed using the semiquantitative 2^−*ΔΔ*Ct^ method. The primer sequences for each gene were synthesized by Wuhan Tianyi Huiyuan Biotechnology Co. (Wuhan, China) (Table 1).

### 2.9. Hematoxylin and Eosin and TUNEL Staining

Paraffin-embedded tissues were subjected to hematoxylin and eosin (HE) and TUNEL staining. For HE staining, the sections were sequentially stained with hematoxylin for 3 min, 1% hydrochloric acid alcohol for 1 min, and 0.5% eosin solution for 3 min. The sections were washed using distilled water for the appropriate time before replacing the staining solution. The sections were soaked in 80% ethanol and 95% ethanol for 15 s, followed by anhydrous ethanol for 3 min. After washing the sections twice with xylene for 5 min each time, the sections were sealed with neutral gum.

For TUNEL staining, the sections were incubated with proteinase K working solution at 21–37°C for 15–30 min. Next, 50 *μ*L of TUNEL working solution (50 *μ*L enzyme solution + 450 *μ*L labeling solution) was added to the samples and incubated in a wet box for 30 min. The sections were rinsed thrice with PBS, restained with hematoxylin, dehydrated, and cleared. The blocked slices were photographed using a microscope.

### 2.10. Statistical Analysis

At least three replicates were evaluated in each experiment. Means were compared using one-way analysis of variance (ANOVA) followed by the William Duncan post hoc test for multiple comparisons (SPSS version 20 software, SPSS, Inc., Chicago, IL, USA). Certain *in vivo* data were analyzed using two-way repeated measures analysis of variance, followed by the Bonferroni post hoc test. When Mauchly's test of sphericity was not satisfied, epsilon correction was applied. Statistical significance was set at *P* < 0.05.

## 3. Results

### 3.1. Identification of Astrocytes

**Figure 1:**
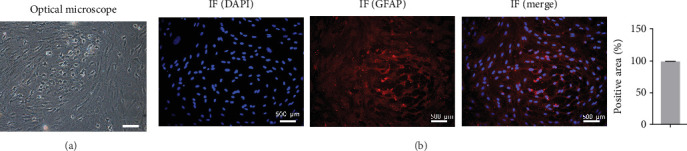
Identification of primary astrocytes using microscopy and immunofluorescence. (a) Cell morphology was observed under an inverted phase-contrast microscope. (b) Detection of glial fibrillary acidic protein expression in an immunofluorescence assay. Scale bar = 500 *μ*m; *n* = 3.

Cell morphology was observed using an inverted phase contrast microscope (Figure 1(a)). The cells were refractive, irregularly shaped, and predominantly polygonal, with large, flat, and abundant cytoplasm. The nuclei were round or ovoid, with 1–2 nucleoli on one side of the cytoplasm. Multiple thick, short-branched primary cytoplasmic protrusions with glial cell growth characteristics were observed. The GFAP immunofluorescence localization results showed that more than 98% of cells in the cytoplasm were stained with GFAP; no staining was observed in the nucleus (Figure 1(b)). These results demonstrate that the extracted cells comprised a relatively pure population of astrocytes and were suitable for use in subsequent experiments.

**Figure 2:**
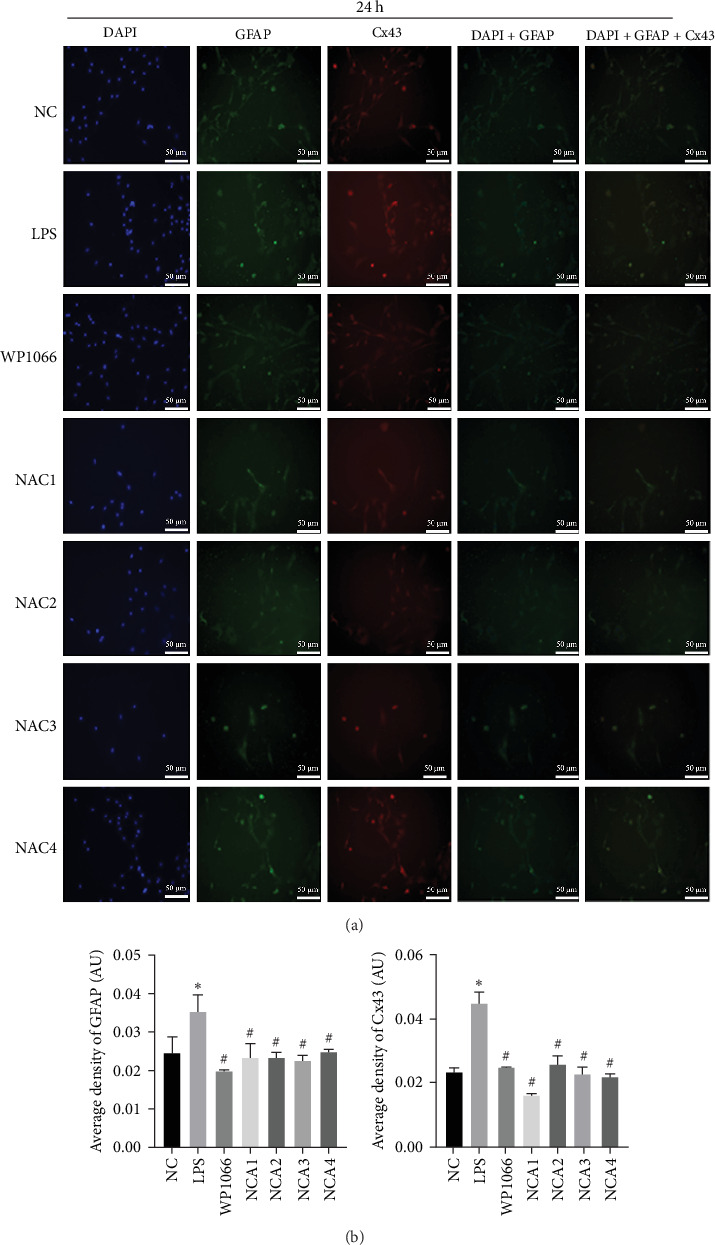
Optimal concentration and time of action of N-acetylcysteine (NAC) for immunofluorescence screening. (a and b) Changes in fluorescence intensity of GFAP and CX43 in each group at 24 hr of NAC treatment. NC, normal control group; lipopolysaccharide (LPS), 1 *μ*g/mL LPS; NAC1, LPS + 5 mM; NAC2, LPS + 2.5 mM; NAC3, LPS + 1 mM; and NAC4, LPS + 0.5 mM. Scale bar = 50 *μ*m; *n* = 3. *⁣*^*∗*^*P* < 0.05 vs. NC group. ^#^*P* < 0.05 vs. LPS group.

**Figure 3:**
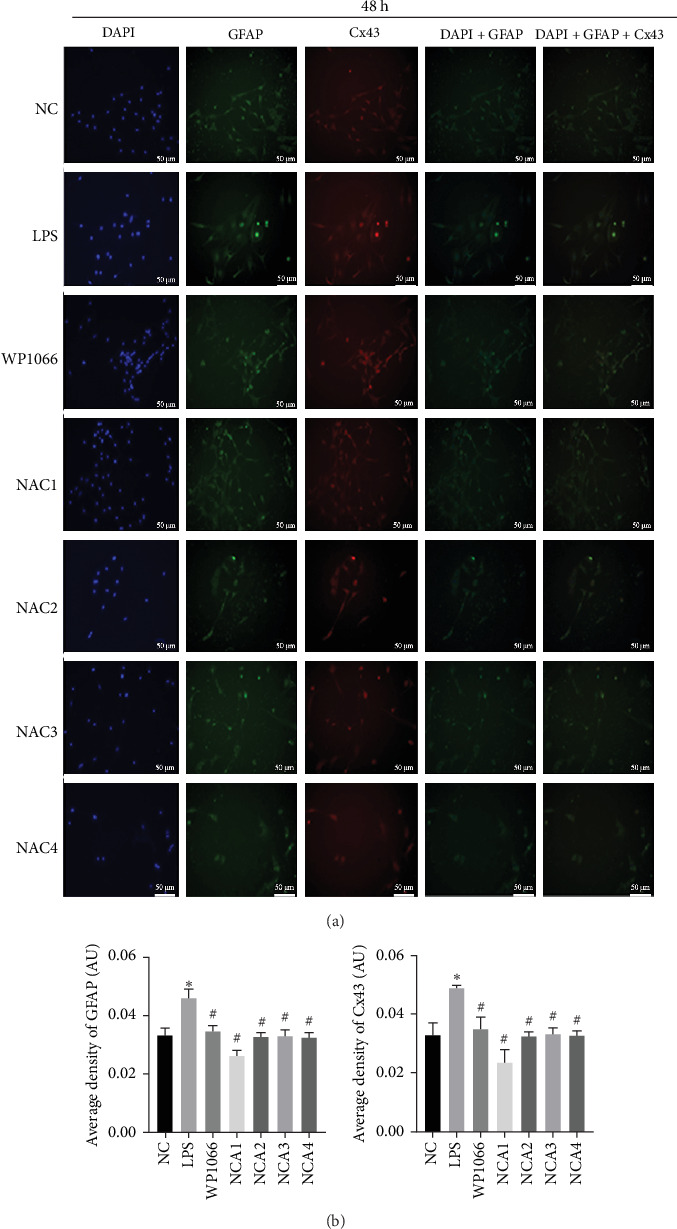
Optimal concentration and time of action of N-acetylcysteine (NAC) for immunofluorescence screening. (a and b) Changes in fluorescence intensity of GFAP and CX43 in each group at 48 hr of NAC treatment. NC, normal control group; lipopolysaccharide (LPS), 1 *μ*g/mL LPS; NAC1, LPS + 5 mM; NAC2, LPS + 2.5 mM; NAC3, LPS + 1 mM; and NAC4, LPS + 0.5 mM. Scale bar = 50 *μ*m; *n* = 3. *⁣*^*∗*^*P* < 0.05 vs. NC group. ^#^*P* < 0.05 vs. LPS group.

Astrocytes were treated with NAC at different concentrations at different times to determine the optimal treatment conditions. Immunofluorescence was used to determine the localization of GFAP and CX43 in the cells. The expression of GFAP and CX43 was upregulated in LPS-treated cells compared with normal cells (Figures 2 and 3), whereas different concentrations of NAC inhibited CX43 expression. Most concentrations of NAC also inhibited GFAP expression; the strongest inhibition of GFAP and CX43 was observed following 5 mM NAC treatment for 48 hr. These two parameters were used in subsequent experiments.

**Figure 4:**
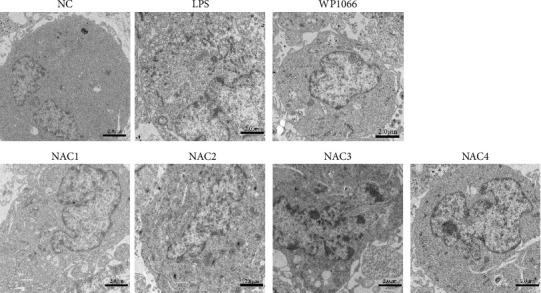
Morphological changes in astrocytes after 48 hr of intervention with different concentrations of N-acetylcysteine (NAC) under a transmission electron microscope. NC, normal control group; lipopolysaccharide (LPS), 1 *μ*g/mL LPS; NAC1, LPS + 5 mM; NAC2, LPS + 2.5 mM; NAC3, LPS + 1 mM; and NAC4, LPS + 0.5 mM. Scale bar = 2.0 *μ*m; *n* = 3.

We observed astrocyte hypertrophy and proliferation following NAC treatment via TEM. LPS significantly increased cell proliferation in the NC group, whereas WP1066 (JAK inhibitor) [34] reversed the proliferation induced by LPS in normal cells (Figure 4). Similarly, high concentrations of NAC significantly inhibited LPS-induced proliferation, which gradually diminished with decreasing NAC concentrations.

### 3.2. NAC Reduces the Expression of Secreted Proteins in Reactive Astrocytes *In Vitro*

**Figure 5:**
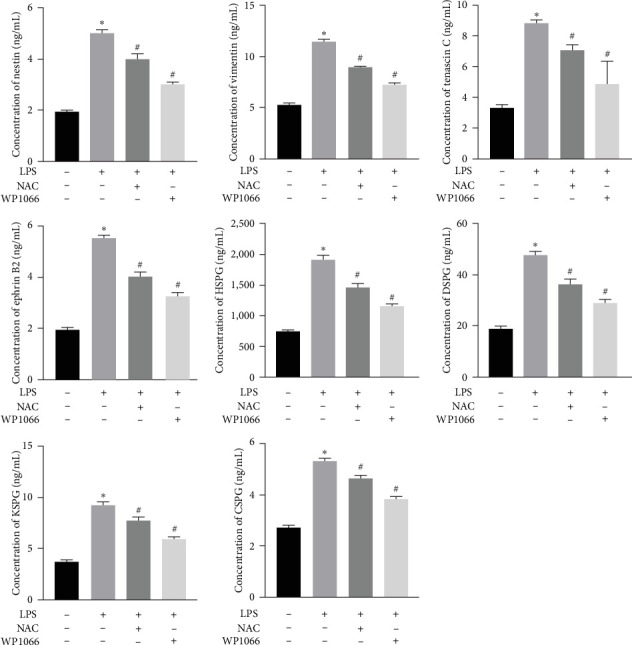
Concentration of secreted proteins (nestin, vimentin, tenascin C, ephrin-B2, HSPG, DSPG, KSPG, and CSPG) detected using ELISA. Data are shown as the mean ± SD (*n* = 3). *⁣*^*∗*^*P* < 0.05 vs. control group. ^#^*P* < 0.05 vs. LPS group.

To investigate the effect of NAC on secreted proteins in a model of LPS-induced reactive astrocytes *in vitro*, we examined the expression of nestin, vimentin, tenascin, ephrin-B2, HSPG, DSPG, KSPG, and CSPG proteins using ELISA. LPS significantly increased the expression of these proteins in normal cells, whereas WP1066 reversed these effects. NAC also significantly decreased the expression of these proteins; however, the effect was not as significant as that caused by WP1066 (Figure 5).

### 3.3. NAC Regulates the JAK/STAT Pathway *In Vitro*

**Figure 6:**
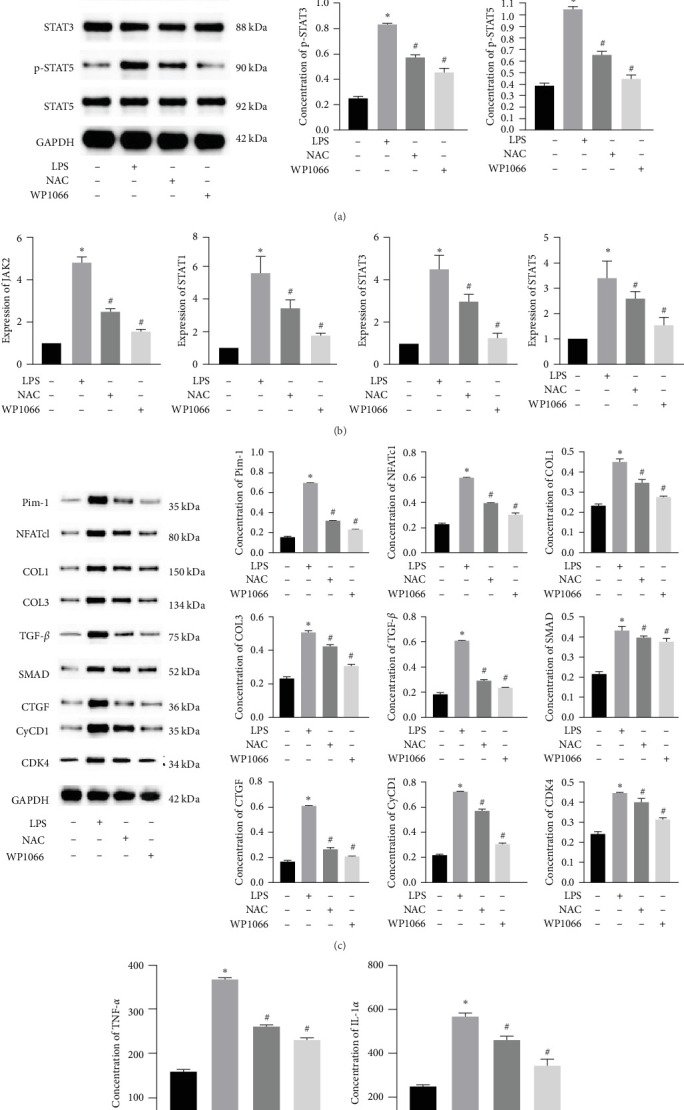
Effect of N-acetylcysteine on JAK/STAT pathway. (a) Phosphorylation level of major proteins (p-JAK2, p-STAT1, p-STAT3, and p-STAT5) on JAK/STAT pathway detected using western blotting. The quantities were normalized to total levels. (b) Expression of JAK2, STAT1, STAT3, and STAT5 measured using quantitative reverse transcription-PCR. (c) Related proteins downstream of JAK/STAT pathway detected using western blotting. (d) Levels of IL-1*α* and TNF-*α* detected using ELISA. Data are shown as the mean ± SD (*n* = 3). *⁣*^*∗*^*P* < 0.05 vs. control group. ^#^*P* < 0.05 vs. LPS group.

The JAK/STAT pathway and downstream proteins of the pathway were detected via western blotting. The abundances of p-JAK2, p-STAT5, p-STAT3, and p-STAT1 were significantly upregulated in cells after LPS induction. Meanwhile, WP1066 and NAC reversed these effects (Figure 6(a)). Additionally, the expression of *Jak2*, *Stat5*, *Stat3*, and *Stat1* mRNA was significantly increased in cells after LPS induction. These effects were referenced in the WP1066 and NAC groups (Figure 6(b)).

To investigate the effect of NAC on related physiological phenomena, we assessed the expression of proteins downstream of JAK/STAT that are associated with vascular remodeling (PIM1 and NFATc1), cellular fibrosis (COL1, COL3, TGF-*β*, SMAD1, and CTGF), and cell proliferation hypertrophy (CyCD1 and CDK4) proteins. We found that the expression of PIM1 and NFATc1 was significantly increased by LPS, whereas WP1066 and NAC significantly reduced this effect induced by LPS. Moreover, LPS effectively increased cellular fibrosis-associated and proliferation hypertrophy-associated protein abundance in normal cells, whereas WP1066 and NAC reversed these effects (Figure 6(c)).

### 3.4. NAC Downregulates the Expression of Inflammatory Factors IL-1*α* and TNF-*α* in Cells

To investigate the effect of NAC on inflammatory factors in astrocytes, we performed ELISA to assess the expression of IL-1*α* and TNF-*α*. LPS significantly increased the IL-1*α* and TNF-*α* concentrations in cells, whereas WP1066 and NAC attenuated this stimulatory effect of LPS (Figure 6(d)).

### 3.5. SCI Model Construction

**Figure 7:**
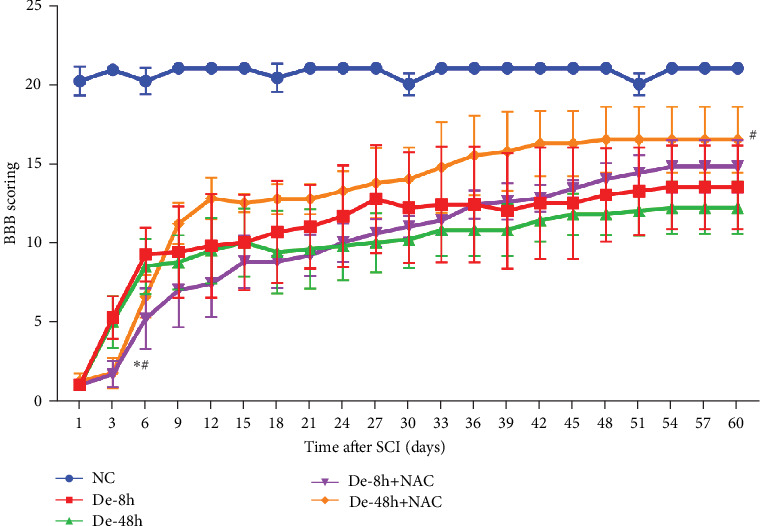
Basso–Beattie–Bresnahan scoring. Data are shown as the mean ± SD (*n* = 10). *⁣*^*∗*^*P* < 0.05 vs. De-8h, and ^#^*P* < 0.05 vs. De-48h. De-8h, decompression therapy (De) post-SCI 8 hr; De-48h, De post-SCI 48 hr; De-8h+NAC, NAC injection after De-8h; NAC was injected at a dose of 50 mg/kg every other day for 2 weeks; De-48h+NAC, NAC injection after De-48h; and NAC was injected at a dose of 50 mg/kg every other day for 2 weeks. The following was also the same as above.

To determine whether model construction was successful, we calculated the BBB score to assess rat motor function. Paralysis was observed immediately in the experimental group, whereas no paralysis was observed in the control group. Additionally, on day 3, the BBB scores of the two groups injected with NAC at 8 and 48 hr after decompression treatment (i.e., De-8h+NAC and De-48h+NAC) were significantly lower than those of the two groups without NAC injection. However, at 60 days, the motility of rats in the De-48h+NAC group was significantly higher than in the other three groups and significantly lower than in the normal group. Although the BBB score in the De-8h+NAC group was higher than in the two groups without NAC injection, the differences were not significant (Figure 7).

### 3.6. NAC Reduces Histopathological Changes and Inhibits Glial Scarring

**Figure 8:**
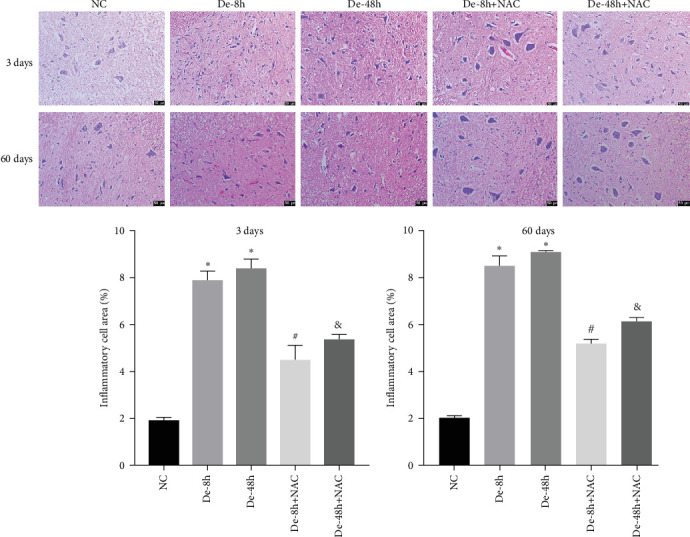
Pathological changes in spinal cord injury were detected using hematoxylin and eosin staining (*n* = 10). Scale bar = 50 *μ*m. *⁣*^*∗*^*P* < 0.05 vs. NC group; ^#^*P* < 0.05 vs. De-8h group; and ^&^*P* < 0.05 vs. De-48h group.

**Figure 9:**
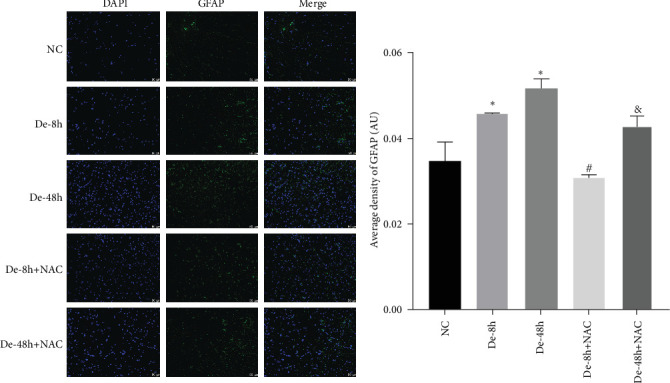
Localization of glial fibrillary acidic protein using immunofluorescence on day 3. Scale bar = 50 *μ*m (*n* = 10). *⁣*^*∗*^*P* < 0.05 vs. NC group; ^#^*P* < 0.05 vs. De-8h group; and ^&^*P* < 0.05 vs. De-48h group.

**Figure 10:**
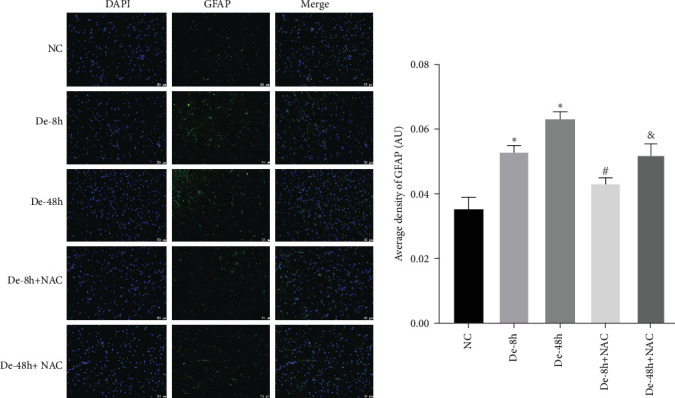
Localization of glial fibrillary acidic protein determined using immunofluorescence on day 60. Scale bar = 50 *μ*m (*n* = 10). *⁣*^*∗*^*P* < 0.05 vs. NC group; ^#^*P* < 0.05 vs De-8h group; and ^&^*P* < 0.05 vs. De-48h group.

**Figure 11:**
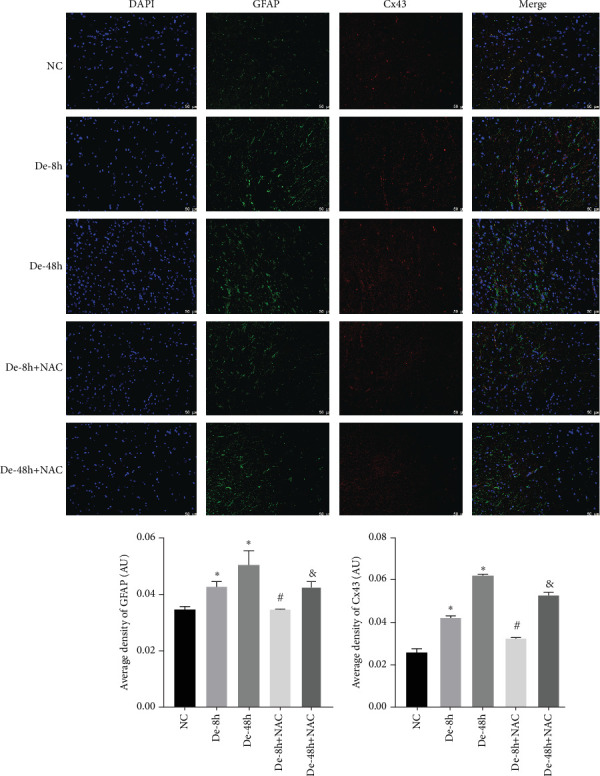
Localization of glial fibrillary acidic protein and connexin 43 determined using immunofluorescence on day 3. Scale bar = 50 *μ*m (*n* = 10). *⁣*^*∗*^*P* < 0.05 vs. NC group; ^#^*P* < 0.05 vs. De-8h group; ^&^*P* < 0.05 vs. De-48h group.

**Figure 12:**
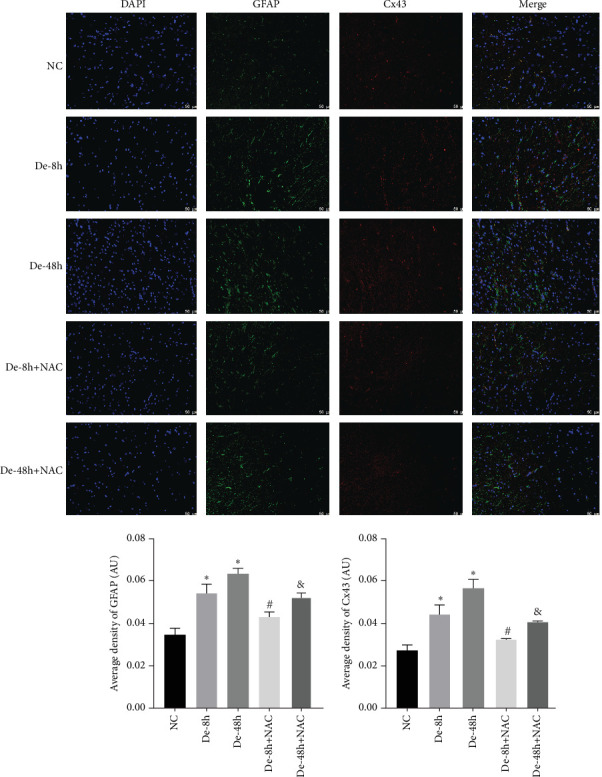
Localization of glial fibrillary acidic protein and connexin 43 determined using immunofluorescence on day 60. Scale bar = 50 *μ*m (*n* = 10). *⁣*^*∗*^*P* < 0.05 vs. NC group; ^#^*P* < 0.05 vs. De-8h group; and ^&^*P* < 0.05 vs De-48h group.

To visualize the pathological changes induced by NAC at bone marrow injury points, tissue sections were stained with HE (Figure 8). The results showed that on days 3 and 60, the De-8h group and the De-48h group had a larger number of inflammatory cells infiltrated than the control group, and the spinal cord tissue was seriously damaged. After NAC treatment, it can reduce inflammatory cell infiltration and reduce spinal cord tissue damage. In addition, we located GFAP (Figures 9 and 10) and CX43 (Figures 11 and 12) on days 3 and 60 via immunofluorescence. GFAP expression increased significantly as the SCI time increased. Meanwhile, NAC decreased GFAP and CX43 expression.

### 3.7. NAC Reduces the Abundance of Proteins Secreted by Astrocytes *In Vivo*

**Figure 13:**
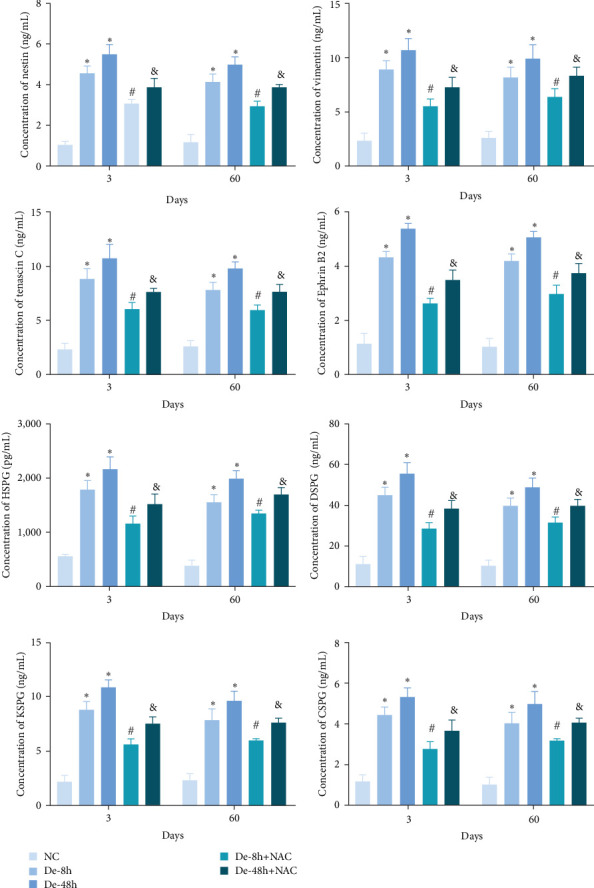
Concentration of secreted proteins (nestin, vimentin, tenascin C, ephrin-B2, HSPG, DSPG, KSPG, and CSPG) *in vivo* detected using enzyme-linked immunosorbent assay. Data are shown as the mean ± SD (*n* = 10). *⁣*^*∗*^*P* < 0.05 vs. NC group; ^#^*P* < 0.05 vs. De-8h group; and ^&^*P* < 0.05 vs. De-48h group.

To verify the effect of NAC on the concentrations of proteins secreted by astrocytes *in vivo*, we performed ELISA to detect the relative abundances of related proteins in the rat serum. SCI increased the expression of secreted proteins (DSPG, HSPG, KSPG, tenascin C, vimentin, CSPG, ephrin-B2, and nestin; Figure 13). While decompression treatment led to a more obvious increase in protein levels, NAC alleviated the upregulation of proteins induced by SCI. The trend in the expression of these proteins was identical at 3 and 60 days.

### 3.8. NAC Regulates the JAK/STAT Pathway *In Vivo*

**Figure 14:**
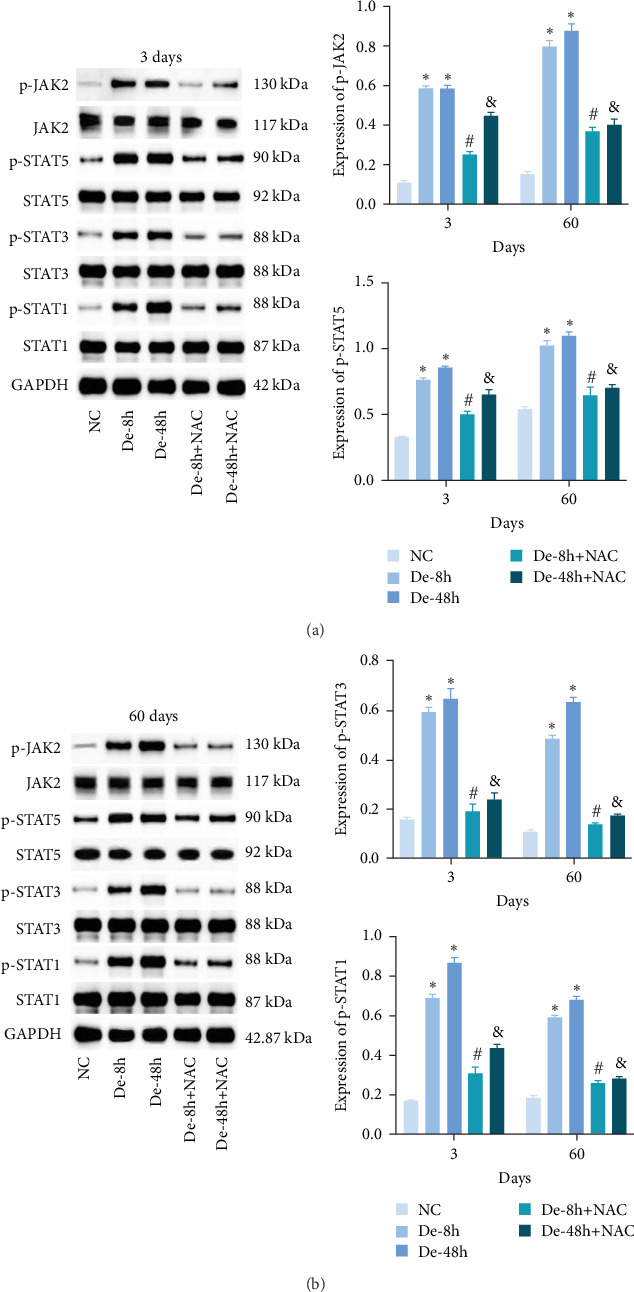
Effect of N-acetylcysteine on JAK/STAT pathway. (a and b) The protein expressions of JAK2, p-JAK2, STAT1, p-STAT1, STAT3, p-STAT3, STAT5, and p-STAT5 *in vivo* were detected by western blotting. Data are shown as the mean ± SD (*n* = 10). The quantities were normalized to total levels. *⁣*^*∗*^*P* < 0.05 vs. NC group; ^#^*P* < 0.05 vs. De-8h group; and ^&^*P* < 0.05 vs. De-48h group.

We also measured the expression of JAK/STAT-related proteins and genes *in vivo* (Figures 14(a) and 14(b)). The expressions of p-JAK2, p-STAT5, p-STAT3, and p-STAT1 were significantly increased in the De-8h and De-48h groups compared with the NC group, decreased in the De-8h+NAC group compared with the De-8h group, and decreased in the De-48h+ NAC group compared with the De-48h group.

**Figure 15:**
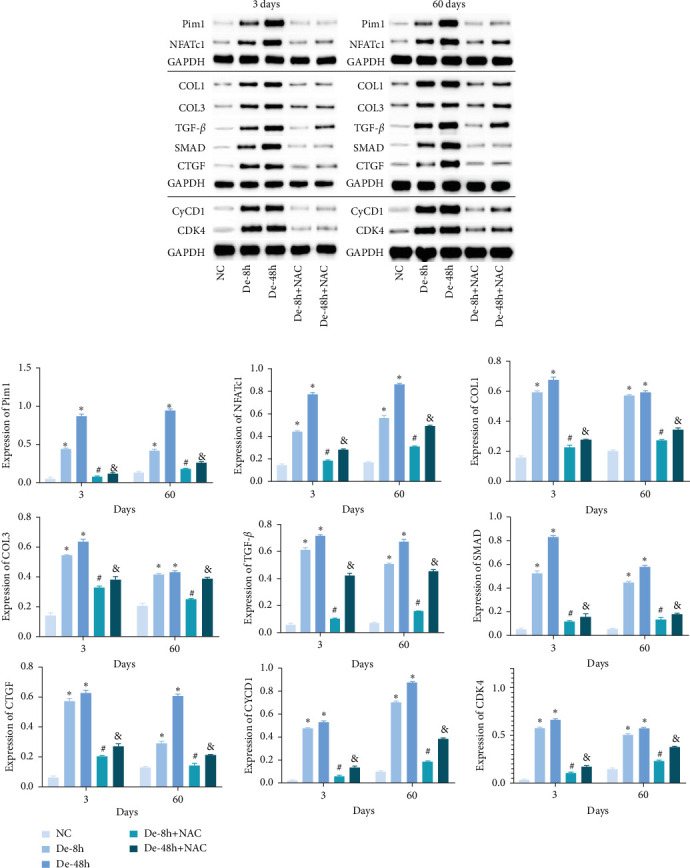
Related proteins downstream of JAK/STAT pathway *in vivo* detected using western blot. Data are shown as the mean ± SD (*n* = 10). *⁣*^*∗*^*P* < 0.05 vs. NC group; ^#^*P*  < 0.05 vs. De-8h group; and ^&^*P*  < 0.05 vs. De-48h group.

Proteins associated with vascular remodeling, cellular fibrosis, cellular proliferation, and hypertrophy were detected *in vivo* using western blotting (Figure 15). The abundance of PIM1, NFATc1, COL1, COL3, TGF-, SMAD1, CTGF, CyCD1, and CDK4 in the De-8h and De-48h groups was significantly higher than in the control group. Excluding PIM1, CyCD1, and CDK4, the effects on other proteins were more obvious at 48 hr than at 8 hr. Nevertheless, these increases were mitigated by NAC, and De-8h+NAC was distinctly more potent than De-48h+NAC alone. On day 60, the expression of all proteins was increased by SCI, particularly in the post-SCI 48-hr group. Similarly, protein overexpression induced by SCI was reversed by NAC, with the most prominent downregulation of all proteins observed after 8 hr of SCI. The abundances of PIM1, NFATc1, COL1, COL3, SMAD1, CTGF, CyCD1, and DK4 were significantly increased at 60 days compared with 3 days. TNF-*α* was significantly decreased overall.

## 4. Discussion

Astrocytes are characterized by cellular hypertrophy during astrocyte proliferation and alterations in the expression of many genes, including GFAP [34]. GFAP is the most widely used immunohistochemical marker for detecting reactive astrocytes [35]. Accordingly, we assessed the morphology and localization of GFAP in cells to confirm that primary astrocytes had been extracted. CX43 has the highest expression in brain astrocytes and is the main channel for coordination between astrocytes. In reactive astrocytes, the expression of CX43 is increased [36]. Although reactive gliosis is a normal physiological response that protects brain cells from further damage, it adversely affects neuronal survival by creating a hostile, impermissible environment for axonal repair [34]. In this study, we determined the optimal intervention concentration (5 *μ*M) and time (48 hr) for NAC treatment by quantifying GFAP and CX43 levels in astrocytes. LPS can promote astrocyte proliferation, and NAC can reverse this proliferation.

Astrocytes release protective or neurotoxic proteins during brain injury. Protective proteins are critical to tissue remodeling, axonal regeneration, glial scar formation, angiogenesis, and neural circuit rearrangement. In contrast, neurotoxic proteins primarily induce neuroinflammation and neuropathology, among other effects [37]. Secreted proteins are closely associated with bone repair under inflammatory conditions [38]. Functionally, secreted proteins such as nestin, vimentin, tenascin, ephrin-B2, HSPG, DSPG, KSPG, and CSPG inhibit neuronal regeneration [39, 40, 41]. Lananna et al. [39] and Xie et al. [42] reported that the expression of secreted proteins, such as nestin, vimentin, and ephrin-B2 [43], is significantly increased following marked GFAP upregulation compared with the NC. In the current study, these secreted proteins were substantially enhanced in LPS-induced reactive astrocytes, whereas NAC downregulated the expression of secreted proteins in these cells. This suggests that NAC promotes neuronal regeneration by inhibiting the expression of secreted proteins. Moreover, De-8h+NAC had the strongest effect on these proteins *in vivo* at 3 and 60 days. Zhao et al. [25] demonstrated the neuroprotective effect of NAC on SCI by evaluating neural function based on the BBB using electromyography. Olakowska et al. [24] also demonstrated the neuroprotective effect of NAC in SCI using the BBB and sciatic functional index. In this study, we obtained the BBB score and determined the proteins secreted by activated astrocytes that inhibit neuronal regeneration.

Qian et al. [6] showed that the mRNA levels of the inflammatory factors IL-6 and TNF-*α* were significantly increased in microglia activated by LPS; additionally, NAC inhibits secreted proteins. According to Tang et al. [44], inhibitors of secreted proteins correct SCI by inhibiting the production of inflammatory factors in the nuclear factor-kappa B pathway. In this study, the levels of the inflammatory factors IL-1*α* and TNF-*α* in cells were markedly increased by LPS, whereas both JAK inhibitor (WP1066) and NAC inhibited these inflammatory factors. Thus, NAC may further mitigate SCI by inhibiting the JAK signaling pathway. We previously showed that NAC induces apoptosis and inflammation [20]. In this study, WP1066 was added to a primary cell-induced SCI model to verify the relationship between the NAC and the JAK signaling pathways, which further elucidated the mechanism of NAC.

Interleukin receptors characterized by JAK family tyrosine kinases regulate the JAK/STAT signaling pathway, which is associated with various diseases such as idiopathic pulmonary fibrosis [45], inflammatory breast cancer [46], and myocardial ischemia reperfusion and play a pivotal role in bone homeostasis and regeneration [47]. Hence, to further examine the mechanism underlying the effect of NAC on SCI, we evaluated proteins related to the JAK/STAT signaling pathway. Al-Samhari et al. [31] showed that NAC remarkably reduced the expression of STAT3 and p-STAT3 in the hippocampus and prefrontal cortex of depressed rats. Similarly, we found that the expression of JAK2, STAT5, STAT3, STAT1, p-JAK2, p-STAT5, p-STAT3, and p-STAT1 was reduced. We further demonstrated that NAC had a regulatory effect on the JAK/STAT pathway.

Many clinical studies [48] have reported that early surgical decompression has a positive impact on the behavioral and pathological outcomes of animal SCI models. A review by Ahuja et al. [1] described a decrease in the incidence of acute hospital complications in patients who undergo early decompression surgery. In this study, we compared the changes in various indicators of NAC treatment after De-8h and De-48h. Our *in vitro* and *in vivo* results are consistent with those of previous studies, showing that NAC treatment after 8 hr of decompression significantly inhibited all indicators. In addition, by comparing days 3 and 60 *in vivo*, we found that the inhibitory effect of NAC on each index was durable, with no tendency for rebound except in individual indices. Kaynar and Hanci reported that MDA levels decreased after NAC treatment [49, 50]. In this study, NAC significantly reduced the levels of GFAP and CX43 during treatment for SCI.

## 5. Conclusion

**Figure 16:**
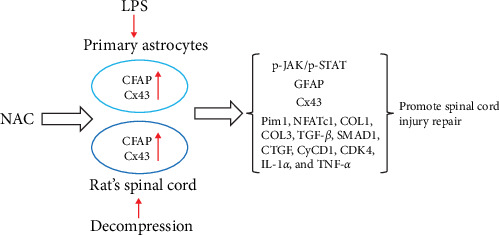
Model of N-acetylcysteine (NAC) treatment of spinal cord injury prepared by inhibiting astrocyte proliferation.

NAC inhibits astrocyte proliferation and GFAP and CX43 expression and downregulates secreted proteins that inhibit neuronal regeneration. Additionally, NAC may inhibit astrocyte proliferation caused by decompression treatment through the JAK/STAT pathway and alleviate SCI injury (Figure 16). In future studies, the mechanism of action employed by NAC will be further elucidated in rescue experiments and clinical samples.

### 5.1. Rigor Statement

This study was conducted in accordance with rigorous scientific standards. The research design was carefully planned, taking into consideration potential confounding factors and biases. Sample size calculations were performed to ensure adequate statistical power. The experimental procedures were conducted with strict adherence to established protocols and guidelines. Quality control measures were implemented at each stage of data collection and analysis. Statistical analyses were performed using appropriate methods, and sensitivity analyses were conducted to assess the robustness of the results. The findings were interpreted based on the current literature, and the limitations and potential sources of bias were acknowledged.

### 5.2. Reproducibility Statement

The experimental procedures and protocols described in this article can be reproduced by following the steps outlined below. The necessary materials and equipment are listed, and detailed instructions are provided to ensure accurate replication of the experiments.

## Figures and Tables

**Table 1:** Primer sequences.

Gene	Sequence	Size (bp)
JAK2-F	TCCCCTGGCTGTCTAT	120
JAK2-R	AACCTGCGGAATCTGT	
STAT5-F	TCCCTGTGGTCGTCATT	171
STAT5-R	TCTGCACCTCAGCCTTG	
STAT3-F	GCCTGGTGTGAACTACTC	289
STAT3-R	GCCACTGATGTCCTTTT	
STAT1-F	CTGGAATGATGGGTGC	195
STAT1-R	TTCTTCGTGTAGGGCT	
GAPDH-F	CAAGTTCAACGGCACAG	138
GAPDH-R	CCAGTAGACTCCACGACAT	

## Data Availability

The datasets generated during and/or analyzed during the current study are available from the corresponding author upon reasonable request.

## References

[1] Ahuja C. S., Nori S., Tetreault L. (2017). Traumatic spinal cord injury—repair and regeneration. *Neurosurgery*.

[2] Eli I., Lerner D. P., Ghogawala Z. (2021). Acute traumatic spinal cord injury. *Neurologic Clinics*.

[3] Chio J. C. T., Wang J., Surendran V. (2021). Delayed administration of high dose human immunoglobulin G enhances recovery after traumatic cervical spinal cord injury by modulation of neuroinflammation and protection of the blood spinal cord barrier. *Neurobiology of Disease*.

[4] Hsieh Y.-L., Tay J., Hsu S.-H. (2021). Early versus late surgical decompression for traumatic spinal cord injury on neurological recovery: a systematic review and meta-analysis. *Journal of Neurotrauma*.

[5] Zhu Y. K., Lu F. T., Zhang G. D., Liu Z. P. (2023). A review of strategies associated with surgical decompression in traumatic spinal cord injury. *Journal of Neurological Surgery Part A: Central European Neurosurgery*.

[6] Qian D., Li L., Rong Y. (2019). Blocking Notch signal pathway suppresses the activation of neurotoxic A1 astrocytes after spinal cord injury. *Cell Cycle*.

[7] Kertmen H., Celikoglu E., Ozturk O. C. (2018). Comparative effects of methylprednisolone and tetracosactide (ACTH(1-24)) on ischemia/reperfusion injury of the rabbit spinal cord. *Archives of Medical Science*.

[8] Okada S., Hara M., Kobayakawa K., Matsumoto Y., Nakashima Y. (2018). Astrocyte reactivity and astrogliosis after spinal cord injury. *Neuroscience Research*.

[9] O’Shea T. M., Burda J. E., Sofroniew M. V. (2017). Cell biology of spinal cord injury and repair. *Journal of Clinical Investigation*.

[10] Fan B., Wei Z., Yao X. (2018). Microenvironment imbalance of spinal cord injury. *Cell Transplantation*.

[11] Fan R., Zhang Y., Botchway B. O. A., Liu X. (2021). Resveratrol can attenuate astrocyte activation to treat spinal cord injury by inhibiting inflammatory responses. *Molecular Neurobiology*.

[12] Hou J., Bi H., Ge Q. (2022). Heterogeneity analysis of astrocytes following spinal cord injury at single-cell resolution. *The FASEB Journal*.

[13] Xu J., Fang S., Deng S. (2023). Generation of neural organoids for spinal-cord regeneration via the direct reprogramming of human astrocytes. *Nature Biomedical Engineering*.

[14] Jain M., Singh M. K., Shyam H. (2021). Role of JAK/STAT in the neuroinflammation and its association with neurological disorders. *Annals of Neurosciences*.

[15] Lee K., Choi J.-O., Hwang A., Bae H. W., Kim C. Y. (2022). Ciliary neurotrophic factor derived from astrocytes protects retinal ganglion cells through PI3K/AKT, JAK/STAT, and MAPK/ERK pathways. *Investigative Opthalmology & Visual Science*.

[16] Wang T., Yuan W., Liu Y. (2015). The role of the JAK-STAT pathway in neural stem cells, neural progenitor cells and reactive astrocytes after spinal cord injury. *Biomedical Reports*.

[17] Song Y., Li X., Liu X., Yu Z., Zhang G. (2022). Calycosin alleviates oxidative injury in spinal astrocytes by regulating the GP130/JAK/STAT pathway. *Journal of Oleo Science*.

[18] Calzetta L., Matera M. G., Rogliani P., Cazzola M. (2018). Multifaceted activity of N-acetyl-l-cysteine in chronic obstructive pulmonary disease. *Expert Review of Respiratory Medicine*.

[19] Monti D. A., Zabrecky G., Kremens D. (2019). N-acetyl cysteine is associated with dopaminergic improvement in parkinson’s disease. *Clinical Pharmacology & Therapeutics*.

[20] Spence J., Chintapenta M., Kwon H. I., Blaszczyk A. T. (2017). A brief review of three common supplements used in alzheimer’s disease. *The Consultant Pharmacist*.

[21] Morley K. C., Baillie A., Van Den Brink W. (2018). N-acetyl cysteine in the treatment of alcohol use disorder in patients with liver disease: rationale for further research. *Expert Opinion on Investigational Drugs*.

[22] Alamdari D. H., Moghaddam A. B., Amini S. (2020). Application of methylene blue -vitamin C -N-acetyl cysteine for treatment of critically ill COVID-19 patients, report of a phase-I clinical trial. *European Journal of Pharmacology*.

[23] Poe F. L., Corn J. (2020). N-acetylcysteine: a potential therapeutic agent for SARS-CoV-2. *Medical Hypotheses*.

[24] Olakowska E., Marcol W., Właszczuk A., Woszczycka-Korczyńska I., Lewin-Kowalik J. (2017). The neuroprotective effect of N-acetylcysteine in spinal cord-injured rats. *Advances in Clinical and Experimental Medicine*.

[25] Zhao X., Zhao X., Wang Z. (2021). Synergistic neuroprotective effects of hyperbaric oxygen and N-acetylcysteine against traumatic spinal cord injury in rat. *Journal of Chemical Neuroanatomy*.

[26] Karalija A., Novikova L. N., Kingham P. J., Wiberg M., Novikov L. N., Yung W.-H. (2012). Neuroprotective effects of N-acetyl-cysteine and acetyl-L-carnitine after spinal cord injury in adult rats. *PLoS ONE*.

[27] Guo X., He J., Zhang R. (2022). N-acetylcysteine alleviates spinal cord injury in rats after early decompression surgery by regulating inflammation and apoptosis. *Neurological Research*.

[28] McCarthy K. D., de Vellis J. (1980). Preparation of separate astroglial and oligodendroglial cell cultures from rat cerebral tissue. *The Journal of Cell Biology*.

[29] Huang W. L., George K. J., Ibba V. (2007). The characteristics of neuronal injury in a static compression model of spinal cord injury in adult rats. *European Journal of Neuroscience*.

[30] Basso D. M., Beattie M. S., Bresnahan J. C. (1995). A sensitive and reliable locomotor rating scale for open field testing in rats. *Journal of Neurotrauma*.

[31] Al-Samhari M. M., Al-Rasheed N. M., Al-Rejaie S. (2016). Possible involvement of the JAK/STAT signaling pathway in N-acetylcysteine-mediated antidepressant-like effects. *Experimental Biology and Medicine*.

[32] Wu X., Li J., Chen C. (2012). Involvement of CLEC16A in activation of astrocytes after LPS treated. *Neurochemical Research*.

[33] O’Malley D., Liston M., Hyland N. P., Dinan T. G., Cryan J. F. (2011). Colonic soluble mediators from the maternal separation model of irritable bowel syndrome activate submucosal neurons via an interleukin-6-dependent mechanism. *American Journal of Physiology-Gastrointestinal and Liver Physiology*.

[34] Pekny M., Wilhelmsson U., Tatlisumak T., Pekna M. (2019). Astrocyte activation and reactive gliosis—a new target in stroke?. *Neuroscience Letters*.

[35] Kovacs G. G. (2018). Cellular reactions of the central nervous system. *Handbook of Clinical Neurology*.

[36] Lagos-Cabré R., Burgos-Bravo F., Avalos A. M., Leyton L. (2019). Connexins in astrocyte migration. *Frontiers in Pharmacology*.

[37] Jha M. K., Kim J.-H., Song G. J. (2018). Functional dissection of astrocyte-secreted proteins: implications in brain health and diseases. *Progress in Neurobiology*.

[38] Lee Y., Park Y. S., Choi N. Y., Kim Y. I., Koh Y. G. (2021). Proteomic analysis reveals commonly secreted proteins of mesenchymal stem cells derived from bone marrow, adipose tissue, and synovial membrane to show potential for cartilage regeneration in knee osteoarthritis. *Stem Cells International*.

[39] Lananna B. V., Nadarajah C. J., Izumo M. (2018). Cell-autonomous regulation of astrocyte activation by the circadian clock protein BMAL1. *Cell Reports*.

[40] Song I., Dityatev A. (2018). Crosstalk between glia, extracellular matrix and neurons. *Brain Research Bulletin*.

[41] Elgebaly M. M. (2020). Ephrin-eph signaling as a novel neuroprotection path in ischemic stroke. *Journal of Molecular Neuroscience*.

[42] Xie C., Shen X., Xu X. (2020). Astrocytic YAP promotes the formation of glia scars and neural regeneration after spinal cord injury. *The Journal of Neuroscience*.

[43] Chen X., Zhang L., Hua F., Zhuang Y., Liu H., Wang S. (2022). EphA4 obstructs spinal cord neuron regeneration by promoting excessive activation of astrocytes. *Cellular and Molecular Neurobiology*.

[44] Tang R., Botchway B. O. A., Meng Y. (2020). The inhibition of inflammatory signaling pathway by secretory leukocyte protease inhibitor can improve spinal cord injury. *Cellular and Molecular Neurobiology*.

[45] Milara J., Hernandez G., Ballester B. (2018). The JAK2 pathway is activated in idiopathic pulmonary fibrosis. *Respiratory Research*.

[46] Stevens L. E., Peluffo G., Qiu X. (2023). JAK-STAT signaling in inflammatory breast cancer enables chemotherapy-resistant cell states. *Cancer Research*.

[47] Zhang W.-Y., Zhang Q.-L., Xu M.-J. (2019). Effects of propofol on myocardial ischemia reperfusion injury through inhibiting the JAK/STAT pathway. *European Review for Medical and Pharmacological Sciences*.

[48] Batchelor P. E., Wills T. E., Skeers P. (2013). Meta-analysis of pre-clinical studies of early decompression in acute spinal cord injury: a battle of time and pressure. *PLoS ONE*.

[49] Hanci V., Kerimoğlu A., Koca K., Başkesen A., Kiliç K., Taştekin D. (2010). The biochemical effectiveness of N-acetylcysteine in experimental spinal cord injury in rats. *Ulusal Travma ve Acil Cerrahi Dergisi—Turkish Journal of Trauma & Emergency Surgery: TJTES*.

[50] Kaynar M. Y., Erdinçler P., Tadayyon E., Belce A., Gümüstas K., Ciplak N. (1998). Effect of nimodipine and N-acetylcysteine on lipid peroxidation after experimental spinal cord injury. *Neurosurgical Review*.

